# A large adrenal myelolipoma: case report and review of the literature

**DOI:** 10.1093/jscr/rjad326

**Published:** 2023-06-07

**Authors:** Iraklis E Katsoulis, Andreas N Dafnis, Chrystalla Sourouppi, Dionysis Katsaounis, E Boti, Niki Arnogiannaki

**Affiliations:** Department of Surgical Oncology, Agios Savvas Oncology Hospital, Athens, Greece; Department of Surgical Oncology, Agios Savvas Oncology Hospital, Athens, Greece; Department of Surgical Oncology, Agios Savvas Oncology Hospital, Athens, Greece; Department of Surgical Oncology, Agios Savvas Oncology Hospital, Athens, Greece; Pathology Department, Agios Savvas Oncology Hospital, Athens, Greece; Pathology Department, Agios Savvas Oncology Hospital, Athens, Greece

**Keywords:** adrenal neoplasm, myelolipoma

## Abstract

Adrenal myelolipoma is a rare benign neoplasm composed of mature adipose tissue and myeloid tissue with a variable amount of hematopoietic elements. Most patients are asymptomatic although some present with pain or even endocrine dysfunction. The rising use of CT and MRI scans has led to an increase of the detection of adrenal myelolipomas in recent years. The indications for surgery are symptomatic patients and lesions bigger than 5 cm or suspicious for malignancy. A case of a 50-year-old woman is presented here who was referred for surgical resection of a large nonfunctioning right adrenal mass. The neoplasm was resected through a midline laparotomy. Histopathology revealed a lesion consisting predominantly of fatty issue containing all types of hematopoietic stem cells and confirmed the diagnosis of myelolipoma.

## INTRODUCTION

Adrenal myelolipoma is a rare benign neoplasm composed of mature adipose tissue and myeloid tissue with a variable amount of hematopoietic elements comprising 3.3–6.5% of all adrenal masses [1]. Adrenal myelolipomas are the second most common adrenal incidentalomas after adrenal adenomas. They have an approximate autopsy prevalence of 0.08–0.2%. In 95% of cases they are unilateral with slight right-sided predilection [2], variable in size, most often found during midlife with a median age of diagnosis around 51 years, and affect both sexes almost equally [1].

Rarely myelolipomas are encountered outside the adrenal glands and are termed as extra-adrenal myelolipoma [3, 4]. The pathogenesis is believed to be either metaplastic changes in the mesenchymal cells or overstimulation by adrenocorticotrophic hormone (ACTH) [1, 5, 6]. Clinically, they usually are asymptomatic and less frequently present with abdominal or flank pain. On imaging, adrenal myelolipomas show pathognomonic features consistent with the presence of macroscopic fat [1, 2, 7, 8]. Surgical resection is indicated in cases of significant growth or hormonal hypersecretion [1, 9].

## CASE PRESENTATION

**Figure 1:**
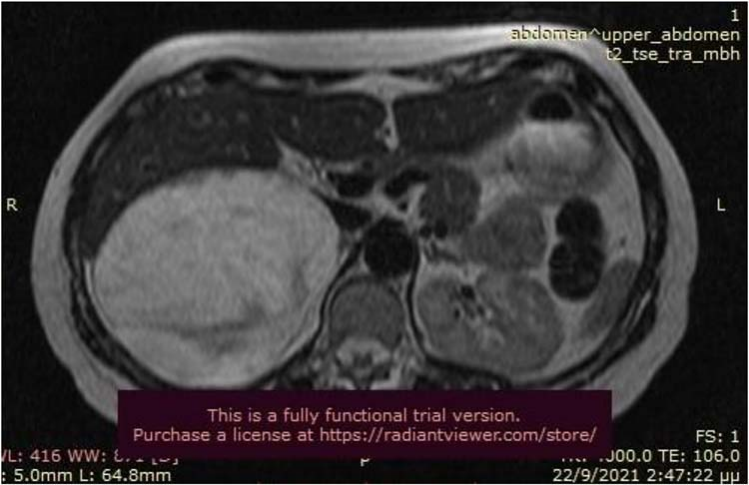
MRI scan depicted a 16 × 15 × 6 cm right adrenal mass with characteristics suggestive of a myelolipoma (transverse plane).

**Figure 2:**
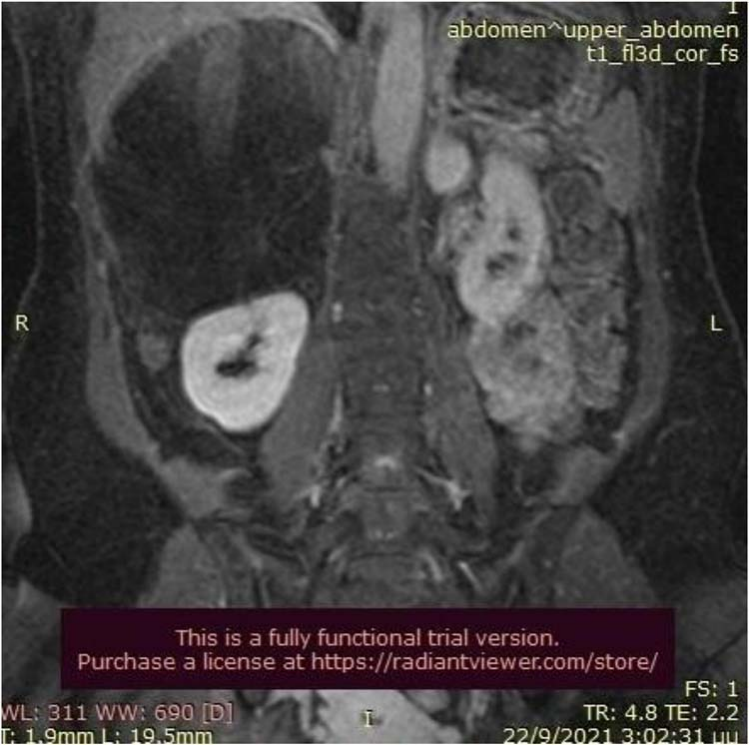
Abdominal MRI scan (frontal plane).

**Figure 3:**
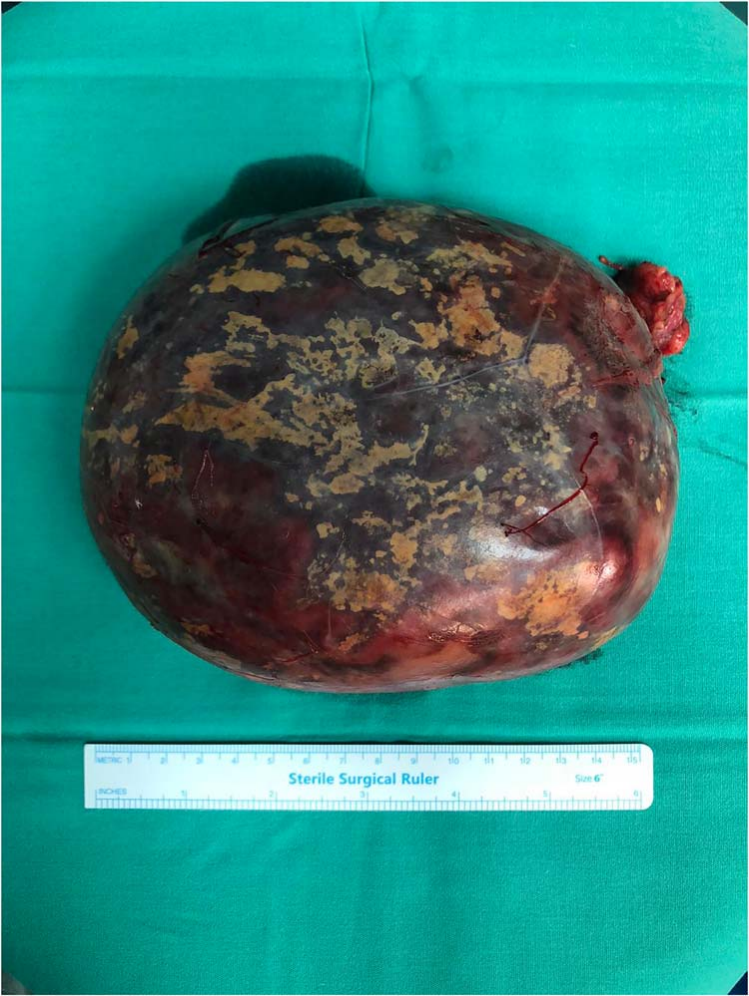
The resected specimen.

**Figure 4:**
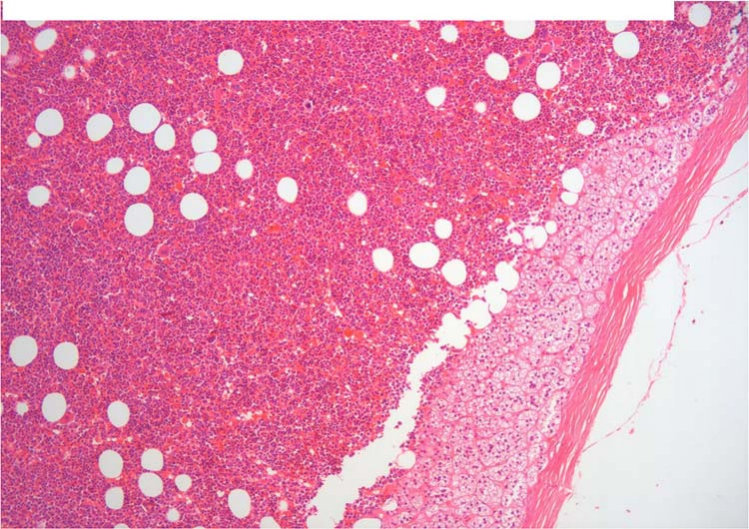
H–E ×200 hematopoietic elements including megakaryocytes with some lipocytes. Above there is a rim of compressed adrenal cortical tissue and fibrous capsule of the adrenal.

**Figure 5:**
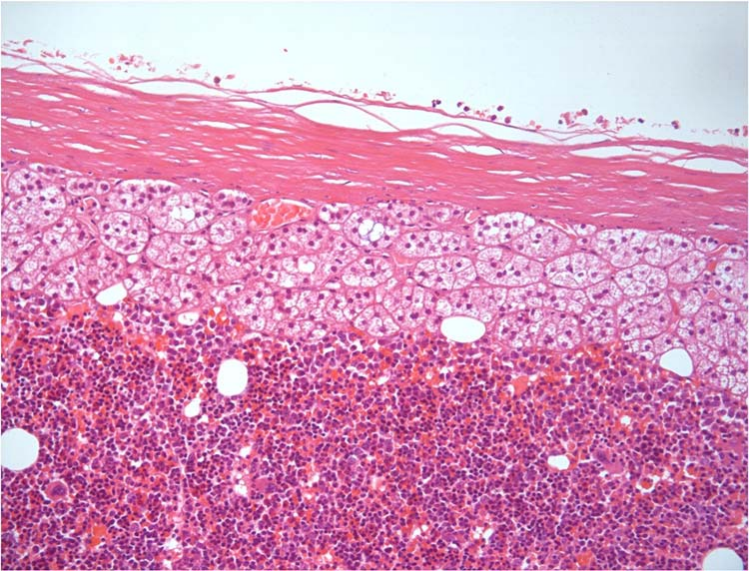
Same histologic features in higher fields.

A 50-year-old woman was referred for surgical resection of a large right adrenal mass, which was incidentally found in an ultrasound scan performed for vague abdominal symptoms. Subsequently  an abdominal MRI scan depicted a 16 × 15 × 6 cm right adrenal mass with characteristics suggestive of a myelolipoma, without evidence of vascular or periadrenal organ invasion (Figs 1 and 2). The patient underwent a comprehensive hormonal serum testing that confirmed a nonfunctioning adrenal mass. Because of the size of the lesion, an open operative approach was selected. Through a supraumbilical midline incision the mass was carefully dissected from the surrounding structures. It was adherent to the right kidney, the liver and the inferior vena cava without, however, compressing it. The harmonic scalper was used and clipping of the adrenal vasculature as appropriate. Intraoperatively, there was no adverse event. The specimen was removed intact and was sent for histological examination (Fig. 3). A silastic corrugated drain was inserted before closure of the laparotomy. The patient’s postoperative course was unremarkable and she was discharged on the third postoperative day. Histopathology revealed a lesion consisting predominantly of fatty tissue containing all types of hematopoietic stem cells and confirmed the diagnosis of myelolipoma (Figs 4 and 5).

## DISCUSSION

Adrenal myelolipomas are benign neoplasms predominantly composed of mature adipose and intermixed myeloid tissue. Myelolipomas are the second most common adrenal incidentalomas after adrenal adenomas. Rarely myelolipomas are encountered outside the adrenal glands. Extra-adrenal sites for myelolipomas include the retroperitoneum, thorax and pelvis. On imaging, adrenal myelolipomas show pathognomonic features consistent with the presence of macroscopic fat. On CT scan, they appear as well-circumscribed, hypodense masses with an attenuation of −90 to −120 HU. The predominantly fatty areas in myelolipoma appear hyperintense on T1 MR images and intermediate to hyperintense on T2 [1, 2, 7, 8]. Etiopathogenesis of these lesions is not definitively known. One hypothesis suggests that stimuli such as necrosis or inflammation could lead to the metaplasia of the reticuloendothelial cells, which could lead to the development of adrenal myelolipomas. Another hypothesis claims that adipocytes develop from the mesenchymal stem cells in the endothelium; this results in inflammation leading the adrenal cortex to secrete mediators responsible for the recruitment of hematopoietic progenitors. Lastly, it was hypothesized that excess ACTH could be responsible for the pathogenesis of adrenal myelolipomas. This theory is supported by the increased incidence of adrenal myelolipomas in congenital adrenal hyperplasia (CAH), where the levels of ACTH can be very high [1, 5, 6]. Patients with CAH exhibit a higher prevalence of adrenal myelolipomas than other patient groups, and are at risk of developing large and bilateral lesions [1]. Adrenal myelolipoma is often associated with conditions like Cushing disease, obesity, hyperlipidemia, hypertension and diabetes, which can be considered as adrenal stimulants. Other theories propose that a stressful lifestyle and an unbalanced diet may also play a role in the natural history of the neoplasm [1, 5, 6].

The size of adrenal myelolipomas is variable and can range from a few millimeters to >10 cm. In the present case, the dimensions of adrenal myelolipoma were 16 × 15 × 6 cm. Myelolipomas occurring in an otherwise normal adrenal gland are the most common pattern of presentation. They are asymptomatic and are incidentally identified during the imaging investigation performed for some other reason. Less commonly, patients present with symptoms such as abdominal, flank or hypochondrial pain and more rarely dyspnea, fever, virilization and weight loss. Large adrenal myelolipomas can cause symptoms of mass effect and occasionally can be complicated by hemorrhage. Isolated larger lesions (typically over 4 cm in size) and those predominantly composed of fat (>50%) have a greater propensity for a hemorrhagic event. In the event of a concomitant adrenal cortical adenoma or hyperplasia, adrenal hormone excess might be detected in patients with adrenal myelolipoma. Patients with CAH exhibit a higher prevalence of adrenal myelolipomas than other patient groups and are at risk of developing large and bilateral lesions. Differential diagnoses based on imaging studies include retroperitoneal lipoma and liposarcoma, upper pole renal angiomyolipoma, retroperitoneal teratoma, adrenal cortical adenoma and adrenal carcinoma. Rarely, there may be difficulty in diagnosing an adrenal myelolipoma by cross-sectional imaging because of low fat content.

Management of adrenal myelolipoma should be decided upon the size of the lesion and the presence of symptoms. Small lesions measuring < 5 cm and those who are asymptomatic are usually monitored via imaging over a period of 1–2 years.

Even asymptomatic tumors need to be removed if are suspicious for malignancy or grow in size during the follow-up period. According to various studies, it is suggested that symptomatic tumors or myelolipomas larger than 5 cm should undergo elective surgical excision. This approach is based on the reported incidence of life-threatening emergencies caused by spontaneous rupture and hemorrhage within large lesions. The operative access depends on the size of the tumor and the expertise of the operator. Mini-invasive and endoscopic techniques are best utilized for smaller-sized lesions. The midline laparotomy could be considered suitable for masses larger than 10 cm or in cases where there are adhesions and infiltration of the surrounding structures. Nevertheless, laparoscopic adrenalectomy has been successfully utilized in some centers even for large myelolipomas [9–11]. Follow-up is mandatory regardless of which surgical method has been employed. There is no evidence of malignant transformation in the literature [1, 5, 8, 9].

On histopathologic examination, myelolipomas are predominantly composed of mature adipose tissue with interspersed hematopoietic tissue components. These fatty elements and hematopoietic areas may be clearly separated, or they are often intermixed.

## CONCLUSION

Most patients are asymptomatic although some present with pain or even endocrine dysfunction. The rising use of CT and MRI scans has led to an increase of the detection of adrenal myelolipomas in recent years. Treatment varies from observation and routine follow-up to surgical excision of the lesion. The indications for surgery are symptomatic patients and lesions bigger than 5 cm or suspicious for malignancy. Although adrenal myelolipoma is rare, physicians should be familiar with this entity and follow the most appropriate treatment.
